# Author Correction: Digit ratios and their asymmetries as risk factors of developmental instability and hospitalization for COVID-19

**DOI:** 10.1038/s41598-022-12900-3

**Published:** 2022-05-23

**Authors:** A. Kasielska-Trojan, J. T. Manning, M. Jabłkowski, J. Białkowska-Warzecha, A. L. Hirschberg, B. Antoszewski

**Affiliations:** 1 Plastic, Reconstructive and Aesthetic Surgery Clinic, Institute of Surgery, Medical University of Lodz, Kopcinskiego 22, 90-153 Lodz, Poland; 2 Applied Sports, Technology, Exercise, and Medicine (A-STEM), Swansea University, Swansea, UK; 3 Department of Infectious and Liver Diseases, Medical University of Lodz, Lodz, Poland; 4 Department of Gynecology and Reproductive Medicine, Karolinska University Hospital, Stockholm, Sweden

Correction to: *Scientific Reports* https://doi.org/10.1038/s41598-022-08646-7, published online 17 March 2022

The original version of this Article contained errors in Table 3, where the mean and SD values were incorrect for “Clinical Composite Asymmetry |Δ2D:4D| + |Δ3D:5D|” for the controls. The incorrect and correct values appear below.

Incorrect:

**Table:** 

Patients			Controls			*p*	*Cohen’s d*
*n*	Mean	SD	*n*	Mean	SD		
**Clinical Composite Asymmetry** │Δ2D:4D│ + │Δ3D:5D│							
49	0.143	0.092	99	0.155	0.109	< 0.0001	**1.04**

Correct:

**Table:** 

Patients			Controls			*p*	*Cohen’s d*
*n*	Mean	SD	*n*	Mean	SD		
**Clinical Composite Asymmetry** │Δ2D:4D│ + │Δ3D:5D│							
49	0.143	0.092	99	0.068	0.046	< 0.0001	**1.04**

The original article has been corrected.

